# An Optimized and Versatile Counter-Flow Centrifugal Elutriation Workflow to Obtain Synchronized Eukaryotic Cells

**DOI:** 10.3389/fcell.2021.664418

**Published:** 2021-04-20

**Authors:** Yongqiang Liu, Bei Nan, Junhua Niu, Geoffrey M. Kapler, Shan Gao

**Affiliations:** ^1^Institute of Evolution and Marine Biodiversity, Ocean University of China, Qingdao, China; ^2^Laboratory for Marine Biology and Biotechnology, Qingdao National Laboratory for Marine Science and Technology, Qingdao, China; ^3^Ministry of Education Key Laboratory of Marine Genetics and Breeding, College of Marine Life Sciences, Ocean University of China, Qingdao, China; ^4^Department of Molecular and Cellular Medicine, Texas A&M University, College Station, TX, United States

**Keywords:** cell cycle, counter-flow centrifugal elutriation, synchronization, *Tetrahymena thermophila*, eukaryotic cells

## Abstract

Cell synchronization is a powerful tool to understand cell cycle events and its regulatory mechanisms. Counter-flow centrifugal elutriation (CCE) is a more generally desirable method to synchronize cells because it does not significantly alter cell behavior and/or cell cycle progression, however, adjusting specific parameters in a cell type/equipment-dependent manner can be challenging. In this paper, we used the unicellular eukaryotic model organism, *Tetrahymena thermophila* as a testing system for optimizing CCE workflow. Firstly, flow cytometry conditions were identified that reduced nuclei adhesion and improved the assessment of cell cycle stage. We then systematically examined how to achieve the optimal conditions for three critical factors affecting the outcome of CCE, including loading flow rate, collection flow rate and collection volume. Using our optimized workflow, we obtained a large population of highly synchronous G1-phase *Tetrahymena* as measured by 5-ethynyl-2′-deoxyuridine (EdU) incorporation into nascent DNA strands, bulk DNA content changes by flow cytometry, and cell cycle progression by light microscopy. This detailed protocol can be easily adapted to synchronize other eukaryotic cells.

## Introduction

Synchronization of cell populations is a powerful tool for studying cell cycle regulated events, such as organelle biogenesis, DNA replication, chromosome segregation and the establishment of epigenetic marks on daughter chromosomes (Kolb-Bachofen and Vogell, 1975; Banfalvi, 2011; Jiang et al., 2014, 2019; Sandoval et al., 2015; Delgado et al., 2017; Li et al., 2020). Many techniques have been established to synchronize cells at specific stages of the cell cycle (Laun et al., 2005; Banfalvi, 2011; Willis and Rhind, 2011; Kothari et al., 2016; Juanes, 2017; Crozier et al., 2018). Most widely used approaches are based on one of two distinct strategies for obtaining a homogeneous cell population: transient cell cycle arrest or physical separation. “Arrest-and-release” approaches include temperature-sensitive cell cycle mutants, inhibitors of DNA synthesis or chromosome segregation, pheromone-induced arrest and nutrient starvation (Breeden, 1997; Banfalvi, 2008; O’Reilly et al., 2012; Delgado et al., 2017). Treated cells are arrested at a particular stage of the cell cycle and then allowed to progress to the next stage synchronously upon release of the block. These manipulations, however, may perturb cell physiology and can alter the behavior of the cell populations in an unpredictable manner (Cooper, 2003; Banfalvi, 2008). Temperature-sensitive mutants have been mostly used in species that can be propagated in the haploid state, such as *Saccharomyces cerevisiae* (Forsburg and Rhind, 2006; Kume, 2016). Physical fractionation is based on differences in cell density and size, fluorescence signal intensity of DNA binding dyes, or antibodies bound to cells. The two most commonly used separation methods are counter-flow centrifugal elutriation (CCE) and fluorescence-activated cell sorting (FACS) (Bauer, 1999; Banfalvi, 2008; Delgado et al., 2017), due to their minimal effect on cell cycle progression.

**FIGURE 1:**
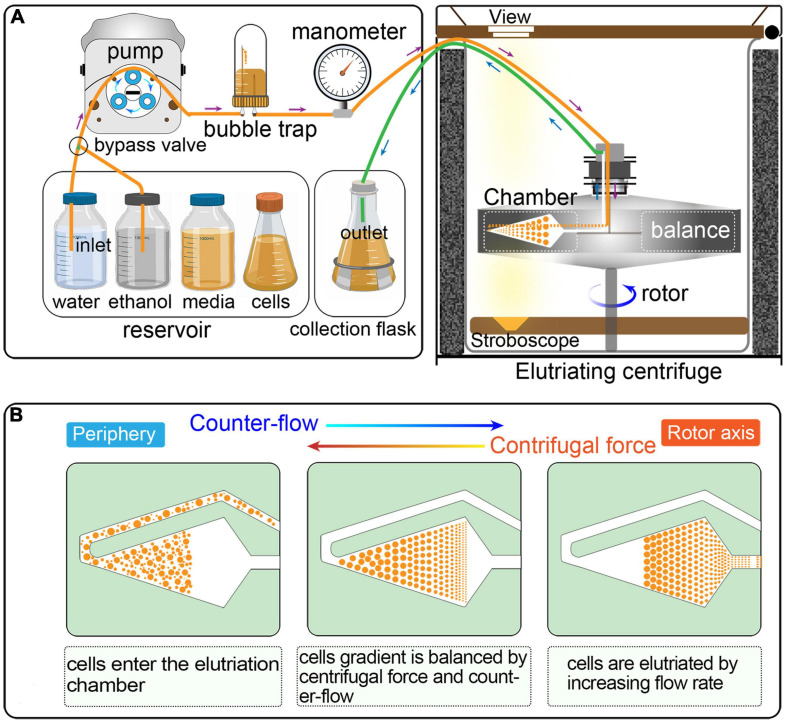
**(A)** Schematic overview of cell synchronization by centrifugal elutriation. The inlet tube is controlled by a three-way valve, which allows the continuous loading of media and/or cells. The bubble trap is half filled with growth medium and the remaining air cushion acts as a damper of the pulsatile flow from the pump. The manometer is placed downstream of the bubble trap. The outlet tube is connected to a collection flask during elutriation. **(B)** Principle of the counter-flow centrifugal elutriation. Cells are separated on the basis of size and density by gradually changing the balance of inward fluid velocity termed “counter-flow” (driving cells toward the axis of rotation) and outward centrifugal force (driving cells away from the axis of rotation).

Counter-flow centrifugal elutriation (CCE) has been widely utilized to synchronized eukaryotic cells (Méndez and Stillman, 2000; Dart et al., 2004; Willis and Rhind, 2011; Ly et al., 2015; Hagan et al., 2016; Horlock-Roberts et al., 2017; Rosebrock, 2017; Crozier et al., 2018). In CCE, cells are separated on the basis of size and density by gradually changing the balance of inward fluid velocity termed as “counter-flow” (driving cells toward the axis of rotation) and outward centrifugal force (driving cells away from the axis of rotation) (Figure 1) (Banfalvi, 2008; Morijiri et al., 2010). CCE produces a uniform gradation of cells of increasing sizes that directly reflects cell cycle progression; smaller cells (in G1) are first eluted followed by larger ones (in S, G2, and in the case of *Tetrahymena*, amitosis). CCE has been applied successfully to study cell cycle-dependent mechanisms in eukaryotes, such as DNA replication, mitosis, cell division checkpoints, transcriptional and translational control, lipid and carbohydrate metabolism, and so on (Brown et al., 1994; Méndez and Stillman, 2000; Dart et al., 2004; Tsubouchi et al., 2013; Blank et al., 2020). Despite its wide application, testing if the reported or calculated parameters are suitable for particular applications or adjusting specific parameters in a cell type/equipment-dependent manner can be challenging.

The ciliate *Tetrahymena thermophila* is a well-known unicellular eukaryotic organism, larger than many mammalian cells (∼30 μm × 50 μm), and has served as an important model organism in a wide range of biological studies including chromosome structure and function, epigenetics, cell biology and cell cycle regulation (Gibbons and Rowe, 1965; Greider and Blackburn, 1985; Brownell et al., 1996; Wang et al., 2017a, b, 2019; Zhao et al., 2017, 2019, 2020; Xu et al., 2019; Sheng et al., 2020). A unique aspect of ciliates is the presence of two physically and functionally distinct nuclei in each cell—the transcriptionally silent diploid “germline” micronucleus (MIC) and the transcriptionally active polyploid (45C) “somatic” macronucleus (MAC) (Coyne et al., 2012; Karrer, 2012; Ruehle et al., 2016). Hence, the vegetative cell cycle culminating in cytokinesis, contains two S phases (MIC and MAC) and two forms of nuclear division-micronuclear mitosis and macronuclear amitosis-the latter of which is coupled to cell division. Various methods have been developed to synchronize the *Tetrahymena* cell cycle, such as homozygous conditional cell cycle mutants, heat shock, starvation and feeding, and drug-induced cell cycle arrest (Cameron and Jeter, 1970; Zeuthen, 1971; Murata-Hori and Fujishima, 1996; Yakisich et al., 2006), each of which has some disadvantages. Whereas heat shock can induce a high degree of synchrony, it also causes abnormal DNA duplication (Zeuthen, 1971; Murata-Hori and Fujishima, 1996). Starvation and feeding has minor side effects, but there is a long S phase lag and the degree of synchrony is suboptimal (Cameron and Jeter, 1970; Murata-Hori and Fujishima, 1996). Microtubule (MT) inhibitors affect multiple physiological processes, but cannot induce the mitotic cell cycle arrest of macronuclear chromosomes which lack centromeres with their corresponding MT attachment sites and randomly segregate by a poorly understood amitotic mechanism (Sedgley and Stone, 1969; Karrer, 2012). The commonly used DNA synthesis inhibitor, hydroxyurea, not only arrests replication fork progression, but also triggers degradation of the *Tetrahymena* Origin Recognition Complex (ORC) (Mohammad et al., 2007; Lee et al., 2015; Sandoval et al., 2015). Other chemical agents induce other deleterious side effects such as the pulverization of micronuclear chromosomes and the formation of extranuclear macronuclear chromatin extrusions bodies (CEBs) (Sedgley and Stone, 1969; Davis et al., 2001; Cole and Sugai, 2012).

Counter-flow centrifugal elutriation has been applied successfully to study cell cycle-dependent mechanisms in *Tetrahymena*, such as the regulation of replication origin licensing and S phase progression (Seyfert et al., 1985; Morrison et al., 2005; Donti et al., 2009; Lee et al., 2015; Sandoval et al., 2015). However, there are still some issues to be addressed for existing protocols, many parameters of which differ significantly from each other (Seyfert et al., 1985; Hengstschläger et al., 1997; Tang et al., 1997; Marsh et al., 2000; Jacob et al., 2001; Cooper, 2003; Donti et al., 2009). Considering the variation in cell size of different *T. thermophila* strains and tetrahymenid species (Seyfert et al., 1984; Zhao et al., 2017; Wang et al., 2019), and the spectrum of available equipment in individual labs, criteria for optimizing parameters are needed.

**FIGURE 2:**
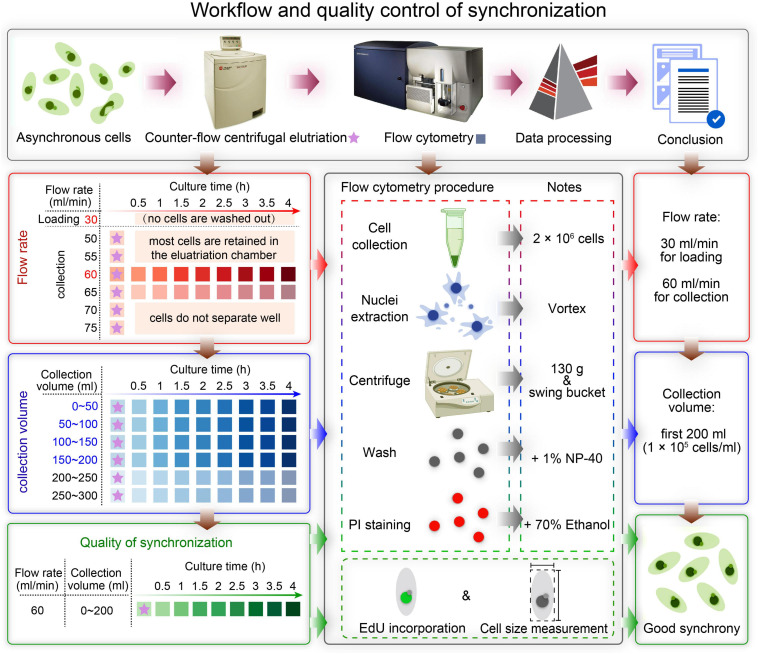
Overview of the workflow and quality control of synchronization protocol. The first row shows a schematic overview of the workflow. The boxes on the left show the three main sampling strategies corresponding to three colors: determination of the optimal flow rate (red), determination of the optimal collection volume (blue), and quality control of synchronization (green). The box in the middle illustrates three ways to assess the degree of synchrony: flow cytometry, EdU incorporation and cell size measurement. The three boxes on the right show the results of each step.

In this study, we describe in detail how to identify optimal conditions for obtaining a large population of synchronized cells, using *Tetrahymena thermophila* as a testing system. The critical variables include loading flow rate, collection flow rate and collection volume (Figure 2). Different factors affecting the outcome of centrifugal elutriation were systematically examined. Using the optimized protocol, we can efficiently collect 2 × 10^7^ *Tetrahymena* cells in G1 phase in less than 2 h from 1.5 × 10^8^ asynchronously growing cells. We assessed cell cycle progression at successive time point after elutriation and demonstrated that elutriated cells divided synchronously for at least one cell cycle. We also provide an improved macronuclear extraction protocol for flow cytometry that significantly reduces nuclear adhesion. The detailed methodology presented here not only can be used to efficiently obtain synchronized *Tetrahymena thermophila* populations, it can also help researchers optimize the conditions for centrifugal elutriation with other species and cell types of interest.

## Materials and Methods

A summarized scheme for the methodology to obtain synchronized eukaryotic cells is shown in Figure 2. See Supplementary Material for a detailed step-by-step protocol.

### Cell Culture

*Tetrahymena thermophila* strain, SB210, was obtained from the *Tetrahymena* Stock Center^1^. Cells were grown in 1.5 L of 2% PPYS medium (2% proteose peptone, 0.003% sequestrene, 0.2% yeast extract; centrifuged before autoclaving for 20 min at 7,000 g to remove most of the undissolved deposits thereby eliminating their interference with elutriation) at 30°C with shaking (150 rpm) until cells reached the log-phase density (1 × 10^5^ cells/ml).

### Counter-Flow Centrifugal Elutriation

Elutriation was performed in an Avanti J-26S XP elutriator equipped with a JE 5.0 rotor and a standard 40 ml large elutriation chamber (Beckman Coulter Inc., California, United States). A variable-speed pump (BT100-1L equipped with a YZII15 pump head, Baoding Longer Peristaltic Pump Co., Ltd., Hebei, China), a bubble trap, a manometer for monitoring back pressure in the rotor, and tubing with three-way valves were employed to route the cell suspension into the chamber (Figure 1).

The optimized protocol for counter-flow centrifugal elutriation of synchronize cells is shown in Supplementary Table 1. Cell integrity was monitored by light microscopy (Olympus SZX16 stereomicroscope, Olympus Co., Japan) at 100 × magnification. Fractions were collected from the elutriation system and nuclei were extracted immediately and stored at −20°C for flow cytometry analysis.

### Flow Cytometry

Flow cytometry was used to assess the cell cycle. For this analysis, 2 × 10^6^ elutriated cells were collected and washed once with 10 mM Tris–HCl (pH 7.4) by centrifugation at 800 g for 2 min. The pellet was then resuspended by adding 1 ml ice-cold nuclei extraction buffer (320 mM sucrose, 5 mM MgCl_2_, 10 mM HEPES, 1% Triton X-100 at pH 7.4). Cells were gently vortexed for 30 s and incubated on ice for 10 min. 100 μl 10% Nonidet P-40 (NP-40, final concentration 1%) was added to nuclei extraction cocktail, and mix gently (without making bubbles) until solution clears. Nuclei were pelleted by centrifugation at 130 g for 5 min and washed twice with nuclei wash buffer (320 mM sucrose, 5 mM MgCl_2_, 10 mM HEPES at pH 7.4). Cold 70% ethanol (1 ml) was added to resuspend the nuclei, which were then washed once with 1 ml cold phosphate buffer saline (PBS). Nuclei were stained with propidium iodide (PI)/RNaseA staining buffer (final concentration: PI 50 μg/ml, RNaseA 100 U/ml; Code No. 550825, BD, Biosciences Co., Shanghai, China). Nuclear adhesion ratio was defined as the number of adhesive MACs divided by the total number of MACs.

The flow cytometer was gated to collect data on the polyploid (45C) macronucleus only. Data acquisition and analysis were performed using Becton Dickinson FACS Aria III flow cytometer (Becton, Dickinson and Company, Pleasanton, CA, United States) and BD FlowJo^TM^ v10.4 software^2^. The fluorescence intensity of DNA content was presented as a logarithmic display (Brunk et al., 1982; Li and Elsasser, 2006; Terry and White, 2006; Li, 2011). The optimized protocol for flow cytometry analysis is shown in Supplementary Table 2.

### Measurement of Cell Number and Cell Size

A Beckman Coulter Z2 Particle Counter was used to count the cell number. To measure the cell size, cells (*n* > 300) were fixed in 2% (w/v) formaldehyde buffer and bright-field microscopy images were obtained using an Olympus BX43 microscope with a DP72 camera at 200 × magnification. Cell length and width were measured by the Olympus software cellSens Dimension v.1.6. Data are presented as mean ± standard deviations.

### EdU Labeling

A 25 ml volume of elutriated cells (1 × 10^5^ cells/ml) was continuously incubated with 100 μM EdU (5-ethynyl-2′-deoxyuridine, Code No. 1149-100, Click Chemistry Tools, Scottsdale, United States) with shaking at 30°C and 2 × 10^5^ cells were collected after 0.25, 0.5, 1, 1.5, and 2 h. Cells were washed once with 10 mM Tris–HCl (pH 7.4) and resuspended in 70% ethanol. After fixation with 2% paraformaldehyde (PFA) in PBS and permeabilization with 1% Triton X-100, cells were incubated with 100 μl Click reaction^TM^ cocktail 10 μl 2 M triethylammonium acetate pH 7.0, 10 μl dimethyl sulfoxide (DMSO, Code No. D8418, Sigma-Aldrich, Shanghai, China), 10 μl premixed 10 mM CuSO_4_ (Code No. 12849, Sigma-Aldrich, Shanghai, China) and 20 mM BTTAA (Code No. 1236-100, Click Chemistry Tools, Scottsdale, United States), 0.24 μl 1.3 mM AF488 Picolyl-Azide (Code No. 1276-1, Click Chemistry Tools, Scottsdale, United States) and 10 μl 200 mM sodium ascorbate (Code No. A7631-25G, Sigma-Aldrich, Shanghai, China) for 1 h in the dark. Cells were mounted with DAPI (Code No. P36935, Thermo Fisher Scientific, Shanghai, China). Images were collected using an Olympus BX43 fluorescence microscope with a DP72 camera at 400 × magnification. Data on the polyploid (45C) macronucleus only were recorded.

## Results

### Optimization of Flow Cytometry for Cell Cycle Analysis

**FIGURE 3:**
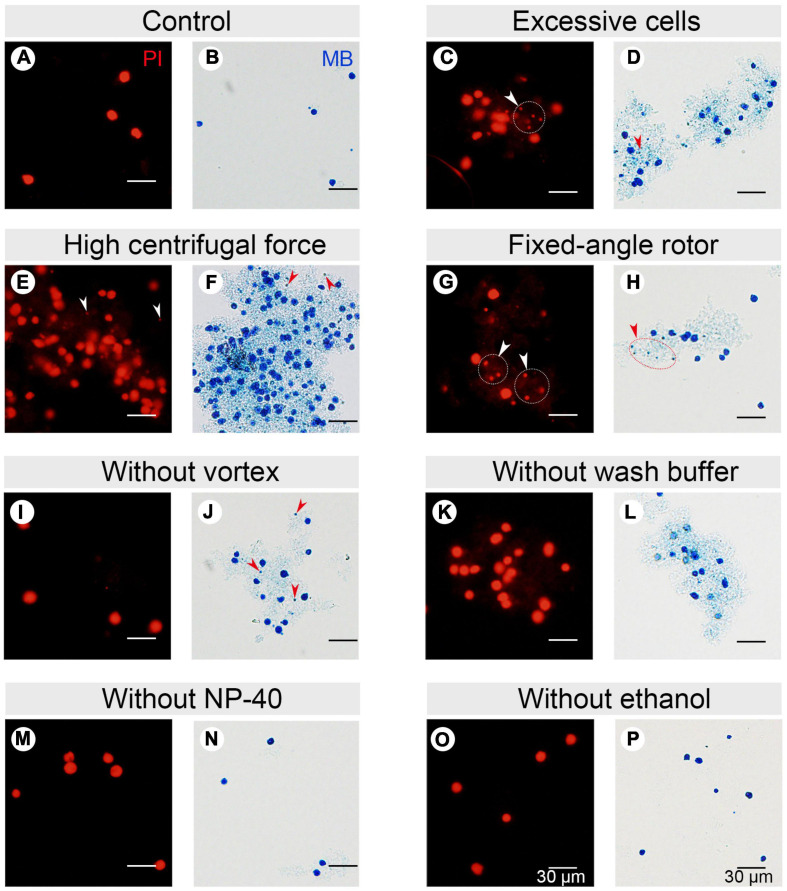
Effect of different factors on macronucleus (MAC) purification for flow cytometry. **(A,B)** Control: MACs purified using the optimized protocol (Refer to Supplementary Table 2). **(C,D)** MACs isolated from excessive cells (1 × 10^7^ cells) (Refer to Supplementary Table 2, #1 change). **(E,F)** MACs collected with high centrifugal force (800 g) (Refer to Supplementary Table 2, #7 change). **(G,H)** MACs collected with a fixed-angle rotor. **(I,J)** MACs collected without vortexing (Refer to Supplementary Table 2, #5 change). **(K,L)** MACs collected without nuclei wash buffer treatment (Refer to Supplementary Table 2, #7 change). **(M,N)** MACs collected without 1% Nonidet P-40 treatment (Refer to Supplementary Table 2, #6 change). **(O,P)** MACs collected without 70% ethanol treatment (Refer to Supplementary Table 2, #10 change). Panels **(A,C,E,G,I,K,M,O)** images are cells stained with propidium iodide (PI), and panels **(B,D,F,H,J,L,N,P)** are cells stained with methylene blue (MB). Arrowheads mark micronuclei (MIC).

In CCE, smaller cells (in G1 phase) are first eluted followed by larger ones (in S, G2, and in the case of *Tetrahymena*, amitosis). The cell cycle progression and synchrony of each elutriated fraction are assessed by flow cytometry, the results of which are affected by the adhesion between nuclei. To reduce nuclei adhesion, we optimized the MAC extraction method by testing multiple factors (Figure 3 and Table 1). The number of lysed cells affected the quality of the purified MACs. Excessive cells would cause inadequate lysis and severe nuclei adhesion (Figures 3C,D). On the other hand, if the number of cells is too low, the extracted nuclei would suffer significant loss during subsequent processing steps and would likely be insufficient for flow analysis. We ultimately used 2 × 10^6^ cells to extract nuclei for flow analysis, as recommended by Brunk and Bohman (1986).

**TABLE 1:** Factors affecting flow cytometry.

Factors	Options	Effects	Nuclear adhesion ratio (%)
Number of cells	>2 × 10^6^ cells (1 × 10^7^ cells)	Inadequate lysis and severe nuclei adhesion	51.47
	= 2 × 10^6^ cells	Minimal lysis and/or nuclear adhesion	9.00*
	<2 × 10^6^ cells (1 × 10^5^ cells)	Nuclei scarcity	N/A
Centrifugal force for nuclei	>130 g (800 g)	Severe nuclei adhesion	63.01
	= 130 g	Minimal lysis and/or nuclear adhesion	9.00*
	<130 g (50 g)	Severe nuclei loss	N/A
Rotor type	Fixed-angle rotor	Severe nuclei adhesion	43.61
	Swing rotor	Good	9.00*
Vortex	−	Inadequate lysis or ineffective separation of macronucleus (MAC) from micronucleus (MIC)	16.75
	+	Good lysis and nuclear separation	9.00*
Nuclei wash buffer	−	Severe nuclei adhesion	65.41
	+	Good	9.00*
1% NP-40	−	Slight nuclei adhesion	9.69
	+	No detectable nuclear adhesion	9.00*
70% Ethanol	−	Severe nuclei adhesion or propidium iodide (PI)/RNase staining buffer cannot enter the nucleus	26.77
	+	No nuclear adhesion, strong PI signals	9.00*

*Optimized condition for MAC purification.

In *Tetrahymena*, the polyploid MAC and the diploid MIC are substantially different in size (∼10 μm and ∼1 μm in diameter, respectively), allowing for separation of MAC from MIC by differential centrifugation (Collins and Gorovsky, 2005; Chen et al., 2016; Cheng et al., 2019). Our results showed that high centrifugation speed or use of a fixed-angle rotor failed to separate MACs and MICs and caused severe nuclei adhesion (63.01% nuclei adhesion with high centrifugation speed (800 g) and 43.21% with fixed-angle rotor) (Figures 3E–H and Table 1), and lower speeds caused excessive loss of nuclei. We therefore recommend centrifugation at 130 g with a swinging bucket rotor to maximize the extraction of *Tetrahymena* MACs for flow cytometry.

Nuclear stabilization is required for quantification of yield and DNA content using DNA staining dyes, such as propidium iodide (PI). When nuclei extraction buffer was added, vortexing the cocktail for 30 s did not affect the integrity of the MAC, but facilitated the separation of MAC from MIC (Figures 3I,J). In addition, an immediate wash step after the lysis with wash buffer significantly reduced nuclear adhesion (9.00% with wash vs. 65.41% without wash) (Figures 3K,L and Table 1). Nonidet P-40 (NP-40) is a mild detergent often used for membrane permeabilization while preserving nuclear integrity (Liu et al., 2014). The addition of NP-40 (1%) only slightly reduced nuclear adhesion (9.00% with NP-40 vs. 9.69% without NP-40) (Figures 3M,N and Table 1) and was included in nuclei extraction buffer as it is routinely used in other protocols (Liu et al., 2014; Chen et al., 2016). 70% ethanol was mainly used to fix cells and increase membrane permeability in flow cytometry to allow the large molecule nucleic acid dye PI to enter the nucleus (Darzynkiewicz et al., 1999). We observed that 70% ethanol also facilitated reduced adhesion of undenatured intracellular proteins to the MAC (Figures 3O,P).

### Optimization of Flow Rate for Obtaining Synchronized Cells

To ensure cells were in good condition and to obtain as many G1-phase cells as possible, we used log-phase *Tetrahymena* cells (1 × 10^5^ cells/ml). In total, 1.5 × 10^8^ cells were loaded for elutriation.

Previous investigations found that increasing the pump speed (controlling the outward centrifugal force) led to much higher reproducibility of separation quality than decreasing the rotation speed (controlling the inward fluid velocity) (Hengstschläger et al., 1997). Therefore, we chose to collect samples with a fixed centrifugal speed (850 rpm/70 g) without damaging the cell integrity as previously recommended (Donti et al., 2009). The balance between the two forces was broken by increasing the flow rate.

**FIGURE 4:**
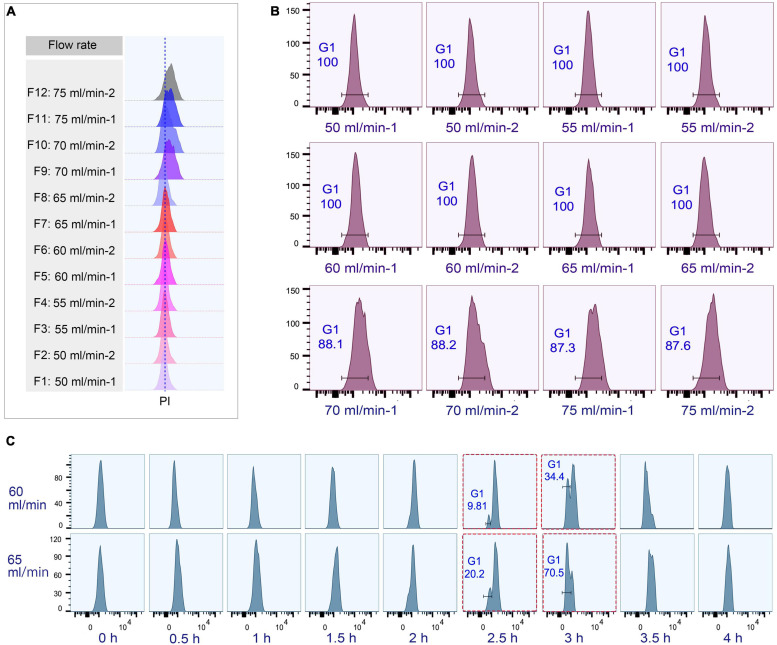
Cell cycle analysis of elutriated *Tetrahymena thermophila* at different collection flow rates. Nuclei were stained with propidium iodide (PI) and analyzed by flow cytometry. **(A)** Flow cytometry profiles of fractions collected at different flow rates (the dotted line demarcates the G1 PI peak). **(B)** Flow cytometry histogram and the percentage of G1 cells of each 50 ml fraction (i.e., 50 ml/min fractions 1 and 2). **(C)** Cell cycle progression of fractions collected at flow rates of 60 ml/min and 65 ml/min. The red boxes highlight that starting populations obtained using different flow rates display a similar percentage of G1 or G2/AM populations at different time points in culture media. The x-axis represents the logarithm of the DNA content (PI fluorescence intensity) and the y-axis represents the number of events (number of events = 10,000).

The loading flow rate was determined to be 30 ml/min because higher flow rate caused cells flowing out of chamber and lower flow rate increased sample loading time. The range of collection flow rate was determined according to published protocols (Kauffman et al., 1990; Hengstschläger et al., 1997; Tang et al., 1997; Marsh et al., 2000; Donti et al., 2009); cells were collected by incrementally increasing flow rates at 5 ml intervals (range 50–75 ml/min), with two 100 ml fractions collected at each rate (Supplementary Table 3). Flow cytometric analysis showed that the flow peaks (corresponding to DNA content) were significantly narrow and on the same straight line in early eluted 50 ml fractions (Fractions 1–8), but were significantly broader and shifted to the higher DNA content in late fractions (Fractions 9–12) (Figure 4A), suggesting that late fractions contained a mixed population of cells at various stages of DNA replication, and hence increased DNA content. This result was further confirmed by the cell cycle phase analysis, showing that early fractions contained more highly enriched G1 populations (100% vs. < 90% in late fractions) (Figure 4B).

To test if the elutriated cells were in good synchrony, cells were cultured and sampled at 30 min intervals for 4 h to trace cell cycle progression. G1 cells eluted at 50 and 55 ml/min rate were in a too low density (about 0.25–0.35 × 10^5^ cells/ml) for subsequent treatment (Supplementary Table 3), and cells at both 70 and 75 ml/min contained a mixed cell population (Figure 4B), so only G1 fractions elutriated at 60 and 65 ml/min were used for further analysis. The cell cycle progression analysis showed that both 60 and 65 ml/min fractions migrated to the higher DNA content synchronously with a narrow peak during 0–2 h and the G1-phase peak re-appeared at 2.5 h (Figure 4C); however, these two fractions clearly differed after 2.5 h with different percentages of G1 phase cells (60 vs. 65: 9.81% vs. 20.2% at 2.5 h; 34.4% vs. 70.5% at 3 h) (Figure 4C), strongly suggesting that they contained cells in different phases. We therefore decided to collect cells at 60 ml/min, because cells collected at 65 ml/min will include mixed populations.

### Optimization of Collection Volume for Obtaining Synchronized Cells

**FIGURE 5:**
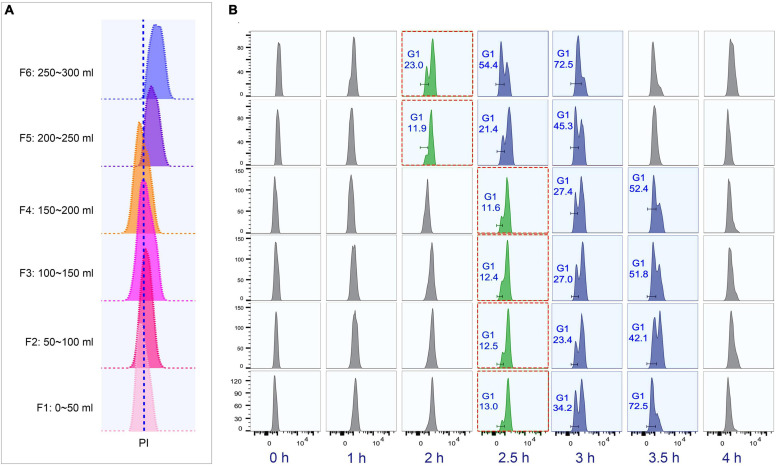
Cell cycle analysis of elutriated *Tetrahymena thermophila* cells of different collection volume. Nuclei were stained with propidium iodide (PI) and analyzed by flow cytometry. **(A)** Flow cytometry profiles of six fractions collected at 60 ml/min flow rate (the dotted line demarcates the G1 PI peak). **(B)** Cell cycle progression of six fractions. The elutriated cells were cultured and sampled at 30 min intervals. The red boxes highlight that different fractions reach a similar state of G1 and G2/Amitosis at different time points. The x-axis represents the logarithm of the DNA content (PI fluorescence intensity) and the y-axis represents the number of events (number of events = 10,000).

To determine the optimal collection volume, a total of six fractions (Fractions 1–6; 50 ml/fraction) were collected at the above-optimized condition (loading flow rate 30 ml/min; rotor speed 850 rpm/70 g; collection flow rate 60 ml/min) (Supplementary Table 4). The results showed that the flow peaks of fractions 1–4 were on the same straight line, while those of fractions 5 and 6 shifted to the higher DNA content (Figure 5A). Moreover, fractions 1–4 shared similar progression kinetics, which were distinct from fractions 5 and 6 (Figure 5B). These results suggested that two types of synchronized populations were present, one in fractions 1–4 and another in fractions 5 and 6. Therefore, the optimal volume was determined to be the first 200 ml. The average cell density of fractions 1–4 was 1 × 10^5^ cells/ml (Supplementary Table 4), so that about 10% of the input material (1.5 × 10^8^ cells) was recovered as G1-phase cells. This is consistent with previous reports (Brunk and Bohman, 1986; Murata-Hori and Fujishima, 1996). Efficient separation of G1 populations could be achieved up to 2 × 10^7^ cells.

### Quality of Cell Synchronization

**FIGURE 6:**
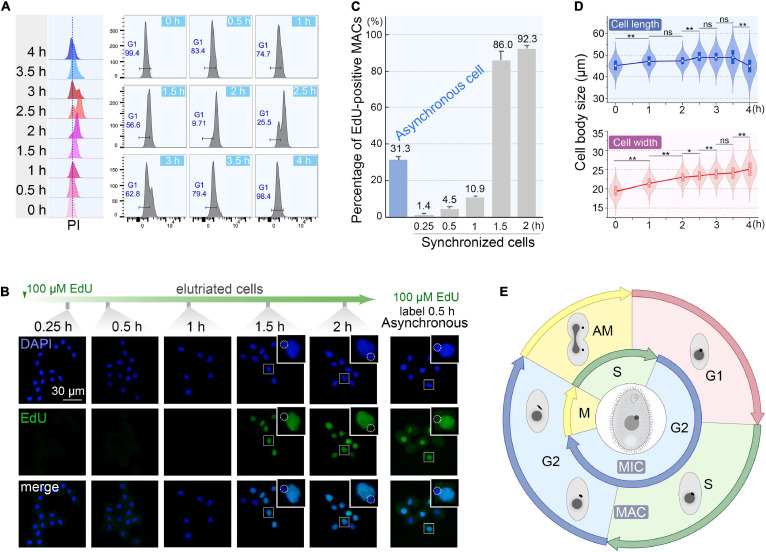
Quality assessment of synchronized cells. **(A)** Cell cycle progression of synchronized *Tetrahymena thermophila*. Histograms show the percentage of G1 cells representing the propidium iodide (PI) signal (x-axis) versus cell count (y-axis) (number of events = 10,000). **(B)** Fluorescent images of synchronous and asynchronous cells labeled by EdU. Synchronized cells were cultured continuously in medium containing 100 μM EdU and sampled at successive time points after elutriation. Asynchronous cells (SB210) were cultured with EdU for 30 min. DAPI staining marks all nuclei (blue signal, exposure time: 0.6 ms). EdU incorporation signal shows macronuclear DNA synthesis (green signal). Note that EdU signals were not detectable in MICs (dotted line circles), which were mostly in the G2 phase. **(C)** Statistical analysis of EdU labeled MACs from panel B. Data are presented as mean ± standard deviations. **(D)** The violin/box plots showing length and width distribution in synchronized cells (n > 300) at different culture intervals post-elutriation. Boxes represent the median and central quartiles; statistical significance was established using Student’s *t*-test. **P < 0.01, *0.01 ≤ *P* < 0.05, ns *P* ≥ 0.05. **(E)** Diagram of the cell cycle of synchronized *Tetrahymena*. The outer circle represents MAC events; the inner circle depicts MIC events. The germline MIC divides by mitosis, whereas the somatic MAC divides by amitosis.

To monitor the quality of synchronization, cells from the first 200 ml collected at 60 ml/min were cultured (Supplementary Table 1) and sampled every 30 min interval to evaluate cell cycle progression by flow cytometry. The flow peaks remained narrow and shifted to the higher DNA content from 0 to 2 h (Figure 6A), with a concomitant reduced percentage of G1 cells (from 100% to <10%) (Figure 6A), indicating that the synchronized G1 cells had entered S phase. The G2/AM flow peak was continuously converted to the G1 flow peak from 2.5 to 4 h (Figure 6A), indicating that the macronuclear chromosomes were undergoing random distribution. After 3.5 h, 98.4% of cells re-entered the G1 phase (Figure 6A) with a gradually increased proportion of G1 cells (from 25.5% to 98.4%), reflecting the amitotic division of macronucleus.

The degree of synchrony was more vividly assessed by EdU labeling (Figures 6B,C). The vast majority of elutriated cells were in G1-phase: 98.6% of the MACs were EdU-negative. Their synchronous transition into S phase occurred from 1 to 1.5 h post elutriation, with EdU-positive MACs increased sharply to 86.0%. In comparison, about 30% of cells in an asynchronously growing population generated EdU-positive macronuclear signals.

The cell size shift also matched well with the cell cycle progression. Compared with the asynchronous growing cells, cells in the initial G1 fraction were significantly smaller (20 μm × 45 μm vs. 30 μm × 50 μm). The average cell length increased from 45.1 to 49.1 μm within 3.5 h (Figure 6D), corresponding to the cell progression into G2 phase (relative to MAC S phase) (Flickinger, 1965; Cole and Sugai, 2012; Lynn and Doerder, 2012). The cell length returned to G1 size at 4 h following cytokinesis (Figure 6D). The change in cell width followed a similar trend, except that cells retained the same width at 4 h.

To demonstrate the reproducibility of our optimized protocol, we performed an independent elutriation. The first 200 ml elutriated cells were in good synchrony as measured by EdU incorporation (Supplementary Figure 1). Together, these results indicate that our optimized method yields highly enriched G1-phase *Tetrahymena* cells that proceed synchronously through at least one cell cycle.

## Discussion

Counter-flow centrifugal elutriation (CCE) is a reliable, effective, and widely-used method to synchronize cells for cell cycle research (Bauer, 1999; Davis et al., 2001; Banfalvi, 2008, 2011; Coulais et al., 2012; Grosse et al., 2012; Kume, 2016; Delgado et al., 2017), but how to optimize the procedure by determining specific parameters for particular applications remains challenging. There are three key parameters that collectively determine the results of elutriation: centrifugal force, collection flow rate, and collection volume. In this study, we used a fixed centrifugal speed (850 rpm/70 g) and varied the flow rate to optimize the balance between inward and outward forces, which was reported to improve the reproducibility of elutriation (Hengstschläger et al., 1997). The initial collection flow rate could be roughly determined using the nomogram or equations provided in the Beckman Coulter JE-5.0 elutriation system instruction manual^3^, but for cells like *Tetrahymena* that are not perfectly spherical, this calculation is not directly applicable. We instead consulted some published methods to determine the range of flow rates (Tang et al., 1997; Jacob et al., 2001; Donti et al., 2009), which were then systematically tested and determined that 60 ml/min worked the best. The collection volume is critical for obtaining the highest possible number of cells for downstream experimental analysis without sacrificing synchrony. We compared the progression kinetics of several 50 ml-fractions and selected the first 200 ml cells that fulfilled both standards.

Previous studies suggest a plethora of criteria to evaluate the quality of synchronization (Cooper, 2004; Banfalvi, 2011; Benz et al., 2017), such that large quantities of synchronized cells are obtained, the initial population has narrow size distribution and uniform DNA content, and synchrony can be maintained for at least one cell cycle (Cooper, 2004; Banfalvi, 2011). In this study, we obtained ∼2 × 10^7^ *Tetrahymena* cells in G1 from a single elutriation, which is sufficient for downstream molecular, biochemical, genomic or proteomic experiments involving time points and replicates (i.e., 400 ml culture at a starting density of 0.5 × 10^5^ cells/ml). We then assessed the synchronization quality of elutriated cells by a combination of several methods. We used flow cytometry to monitor the cell cycle progression by measuring cellular DNA content, which showed that the elutriated cells had narrow peaks corresponding to uniform DNA content and could maintain synchronized growth and division for at least one cell cycle. We also employed the EdU incorporation-staining method to show visually that the vast majority of cells could enter S phase simultaneously, which was further supported by the cell size shift during cell cycle progression. In conclusion, our results indicated that using 850 rpm centrifugal force with a 30 ml/min loading flow rate and collecting the first 200 ml cells at 60 ml/min provided the optimal synchronization quality for *T. thermophila*.

*Tetrahymena*’s amitotic (AM) MAC is characterized by a G1-S-G2-AM cell division cycle, which roughly coincides with the regular G1-S-G2-M pattern seen in mitosis (Figure 6E; Flickinger, 1965; Woodard et al., 1972; Doerder, 1979; Cole and Sugai, 2012). The MIC undergoes more conventional mitosis, though with no apparent G1 interval. MIC S phase and mitosis are temporally out of phase with the MAC nuclear cycle (Figure 6E; Flickinger, 1965; Woodard et al., 1972; Doerder, 1979). The highly synchronized cell populations we can obtain by CCE will allow us to address important questions in *Tetrahymena* biology. Transcriptome and proteome analyses can identify unknown players and pathways related to cell cycle events, such as replication origin licensing, replication timing, cell division control including the respective role of multiple cyclin and cyclin-dependent kinases, oral apparatus biogenesis, etc. (Frankel et al., 1976; Gavin, 1976; Mohammad et al., 2003, 2007; Donti et al., 2009; Lee et al., 2015). It should be noted that the expression levels of some genes are elevated in the G1 elutriated population such as hypoxia genes, but this does not affect cell cycle progression and the cyclic expression profiles of cell cycle regulated genes (GM Kapler, unpublished result). Even so, elutriation is by far the best method for synchronization due to minimal perturbation of cell physiology and high degree of synchrony.

It should be noted that cell size variation within a population and cell shape asymmetry will reduce the effectiveness of separation (Hengstschläger et al., 1997; Banfalvi, 2008, 2017). But with detailed procedures, precautions and solutions, and multiple ways of assessing the synchronization quality, we envision an easy adaption and wide application of our protocol in a broader range of eukaryotic cells to obtain synchronized cells. The highly synchronized cells will allow researchers to address important questions of cell biology, including mechanisms that are conserved across the eukaryotic lineage (i.e., cilia and organelle biogenesis), as well as processes that are unique to the Ciliophora phylum (i.e., amitosis and the temporal uncoupling of MIC and MAC S phases). As for conjugative *Tetrahymena* cells, since they are not symmetrical and their size do not vary a lot during conjugation, the efficiency of separation by CCE would be reduced. Nonetheless, the addition of CCE, if fully optimized, may help to eliminate non-mating cells and improve the purity of stable mating pairs or individual progeny cells. Elutriation can also be applied to investigate kinetic analysis of epigenetic marks, such as DNA N^6^-adenine methylation (6mA) and H3 Lysine 27 mono-methylation (H3K27me1), to reveal the mechanism of their transgenerational inheritance and how they are integrated with cell cycle progression (Jacob et al., 2009; Gao et al., 2013; Zhang et al., 2015; Wang et al., 2017a, b, 2019; Zhao et al., 2017; Cheng et al., 2019; Rzeszutek et al., 2020; Wu, 2020).

## Data Availability Statement

The original contributions presented in the study are included in the article/Supplementary Material, further inquiries can be directed to the corresponding authors.

## Author Contributions

SG and GMK conceived the study. YL, BN, and JN performed the experiments. YL and JN performed the flow cytometry data analysis. YL and SG wrote the manuscript with contributions from all other authors. All authors read and approved the final manuscript.

## Conflict of Interest

The authors declare that the research was conducted in the absence of any commercial or financial relationships that could be construed as a potential conflict of interest.

## Footnotes

1 http://tetrahymena.vet.cornell.edu

2 https://www.flowjo.com/solutions/flowjo

3 https://www.beckmancoulter.com/en

## Supplementary Material

Supplementary Figure 1 Statistical analysis of EdU labeled MACs from an independent elutriation. Experimental settings are identical to those in Figures 6B,C. Data are presented as mean ± standard deviations.
