# Supplementary figures and images for: An Optimized and Versatile Counter-Flow Centrifugal Elutriation Workflow to Obtain Synchronized Eukaryotic Cells
Source: Front Cell Dev Biol. 2021 Apr 20;9:664418. doi: 10.3389/fcell.2021.664418 (PMC8093812; doi:10.3389/fcell.2021.664418)

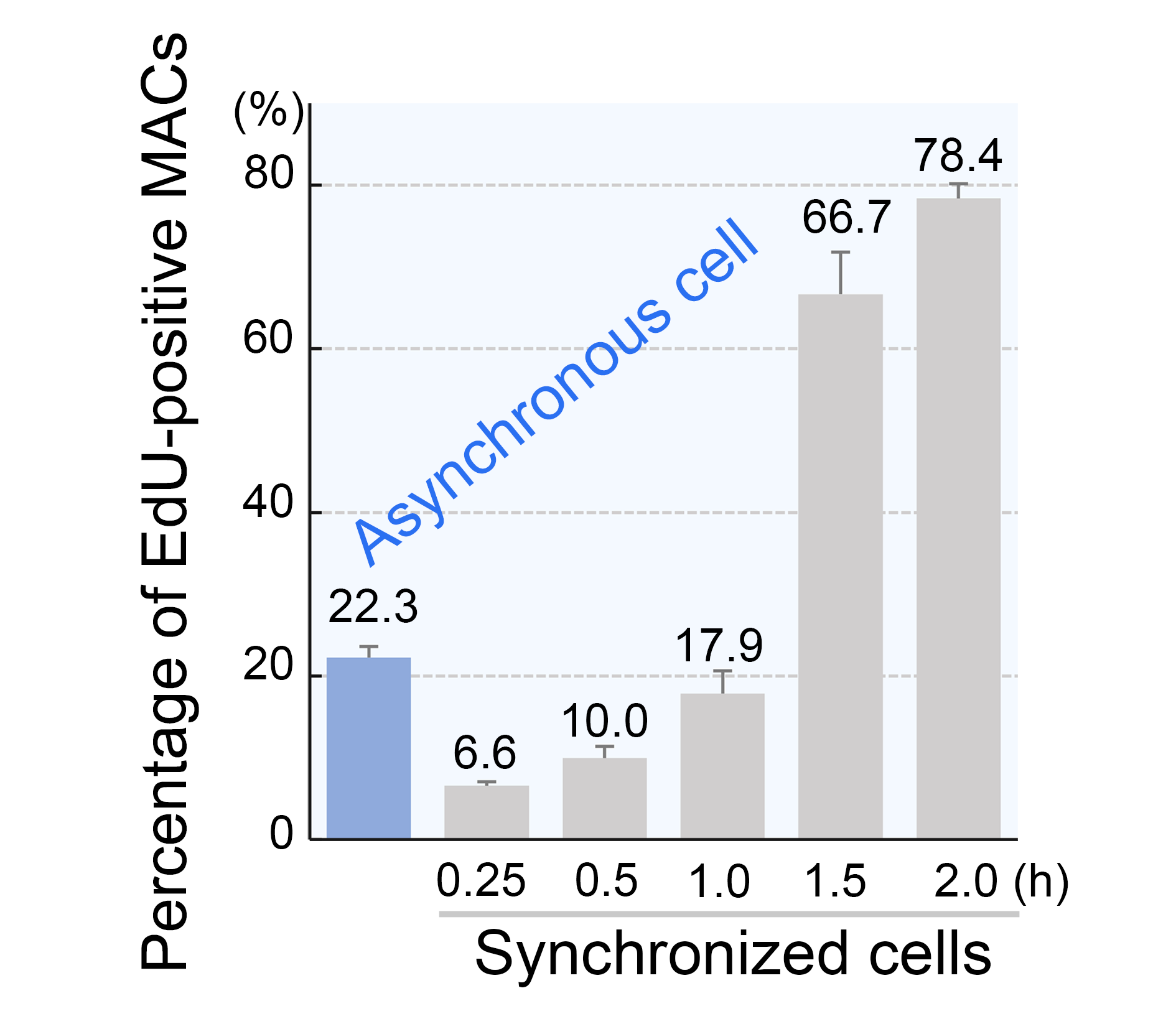

Supplement: Supplementary Figure 1 — Statistical analysis of EdU labeled MACs from an independent elutriation. Experimental settings are identical to those in Figures 6B,C. Data are presented as mean ± standard deviations. [file Image_1.TIF]
