# Supplementary material for: An Optimized and Versatile Counter-Flow Centrifugal Elutriation Workflow to Obtain Synchronized Eukaryotic Cells
Source: Front Cell Dev Biol. 2021 Apr 20;9:664418. doi: 10.3389/fcell.2021.664418 (PMC8093812; doi:10.3389/fcell.2021.664418)
Supplement: Supplementary file 2 [file Table_1.DOC]

**TABLE S****1.** **The optimized elutriation protocol^*^**

| 1. Priming and sterilization of the elutriation system | | | |
| --- | --- | --- | --- |
| Step | | | Note |
| 1) | Place one inlet tube into a bottle containing sterile water, another inlet tube into a bottle containing 10% ethanol, with the outlet tube in a waste beaker. | Set the centrifuge temperature at 25°C, the centrifuge time as hold, the acceleration and the deceleration as max. | |
| 2) | Turn the pump on and allow the sterile water to pass through the elutriation system at 30 ml/min until it flows out of the outlet tube. | Check the bubble trap for any leaks and invert the bubble trap until half-filled with medium. Calibrate the pump by checking the volume of fluid pumped in 1 min. | |
| 3) | Start centrifugation at 850 rpm (70 g) to release bubbles and backpressure and keep the sterile water circulating in the system for 5 min. | Turn off the centrifuge once the rotor speed reaches 850 rpm to confirm whether bubbles are gone. If there are bubbles in the elutriation system, increase flow rate or centrifugal force to eliminate bubbles. | |
| 4) | Turn the three-way valve to 10% ethanol and sterilize the system with 500 ml ethanol at 30 ml/min. | The elutriation system including all tubes should be filled with the 10% ethanol to prevent contamination of the cell sample. | |
| 5) | Turn the three-way valve to allow the sterilized water to rinse the elutriation system at 30 ml/min for at least 20 min and place another inlet tube into a bottle containing elutriation buffer (2% PPYS medium). | Rinse the elutriation system completely, especially the bubble trap, as *Tetrahymena* cells are highly sensitive to ethanol. | |
| 6) | Turn the three-way valve to load about 200 ml elutriation buffer until water is completely flushed out. | Watch for and clear any bubbles in the whole system. | |
| 7) | Remove small bubbles in the inlet tube and adjust the stroboscope to visualize the elutriation chamber through the viewing window of the door. | Gently tap the inlet tube until all bubbles are cleared. Press the strobe lamp power button and slowly turn the knob until the chamber is clearly visible. | |
| 2. Loading cells into the elutriation system and collection of fractions | | | |
| Step | | Note | |
| 8) | Place another inlet tube into a bottle containing log-phase cells and turn the three-way valve to load 1.5 L log-phase cells (1 × 10^5^ cells/ml) into the elutriation system. | Do not stop the pump while the centrifuge is running. Check the eluent under the microscope to make sure there are no intact cells within. | |
| 9) | Once all cells had entered the elutriation chamber, turn the three-way valve to load about 200 ml elutriation buffer into the system. | Load more elutriation buffer to reach complete equalization. | |
| 10) | Increase the pump speed from 30 ml/min to 60 ml/min and collect 200 ml elutriated cells once the liquid in the outlet tube becomes cloudy (about 1 min after the flow rate reaches 60 ml/min). | Prepare enough elutriation buffer (about 1 L) and replenish if necessary. | |
| 11) | Measure the cell density and culture cells in a 20 cm Petri dish and perform successive culture in a 30°C incubator. | Use 20 cm Petri dish to culture cells in a 30°C incubator to allow cells to resume cell cycle progression quickly. | |
| 12) | To finish elutriation, turn off the centrifuge and let sterilized water drive out residual elutriation buffer from the elutriation chamber at 80 ml/min flow rate. Rinse the elutriation system completely with 10% ethanol and 70°C hot water. | Completely remove debris and contaminants from the chamber wall that may negatively affect later runs. | |

*****The elutriation protocol is optimized according to Banfalvi (2017), Banfalvi (2008) and Delgado et al. (2017).

**TABLE S2.** **The optimized flow cytometry protocol**

| The optimized flow cytometry protocol | |
| --- | --- |
| 1 | Collect cells (1–2 × 10^6^ cells/ml) by centrifugation at 800 g for 2 min (Eppendorf Centrifuge 5910R with a swinging bucket rotor). |
| 2 | Wash cells once with 10 mM Tris-HCl (pH 7.4), centrifuge at 800 g for 2 min, then remove supernatant completely. |
| 3 | Resuspend cells in 1 ml cold nuclei extraction buffer (320 mM sucrose, 5 mM MgCl_2_, 10 mM HEPES, 1% Triton X-100 at pH 7.4). |
| 4 | Transfer cells to 1.7 ml low-retention Eppendorf tubes (Axygen® 1.7 mL Maxymum Recovery® snaplock microcentrifuge tube, Code No. MCT-175-L-C, Corning Co.). |
| 5 | Gently vortex cells for 30 sec to facilitate the separation of nuclei from lysate without destroying the nuclei and incubate on ice for 10 min. |
| 6 | Add 100μl 10% Nonidet P-40 (NP-40, final concentration 1%), and invert the tube to mix gently (without making bubbles) until solution clears. |
| 7 | Pellet nuclei by centrifugation at 130 g for 5 min and washed twice with nuclei wash buffer (320 mM sucrose, 5 mM MgCl_2_, 10 mM HEPES at pH 7.4). |
| 8 | Remove as much supernatant as possible with 1 ml pipettor tip; tap the tube to resuspend nuclei in residual buffer. |
| 9 | Check the nuclei yield and integrity after methylene blue (MB) staining with a hemocytometer (Bright-line hemocytometer, Hausser scientific Co., Code No. 3200). |
| 10 | Pre-cool ethanol at -20℃ and tap tubes while slowly adding 1 ml cold ethanol to resuspend the nuclei. |
| 11 | Leave nuclei on ice for 30 min if the sample is to be analyzed immediately, or store at -20°C until needed. |
| 12 | For analysis, keep samples on ice throughout the preparation; spin down nuclei using a swinging bucket rotor at 130 g for 5 min, then remove supernatant completely. |
| 13 | Stain nuclei with 500 μl propidium iodide (PI)/RNaseA staining buffer for 10 min at room temperature (25°C). |
| 14 | Collect nuclei using a swinging bucket rotor at 130 g for 5 min and remove as much supernatant as possible with a 1 ml pipettor tip. |
| 15 | Wash nuclei once with 1 ml cold phosphate buffer saline (PBS) to remove residual staining buffers and reduce false-positive counts in flow cytometry, pellet in a swinging bucket rotor at 130 g for 5 min at 4°C, and remove as much supernatant as possible with a large orifice tip. |
| 16 | Resuspend nuclei in 1 ml cold PBS. |
| 17 | Filter samples with 40 μm cell strainer (Corning® 40 µm cell strainer, Corning Co., Code No. 431750) before flow cytometry to reduce nuclei adhesion. |
| 18 | Perform cell cycle analysis in a multibeam flow cytometer; excite PI with an argon ion laser at 535 nm. |

**TABLE S3. Cell density and flow cytometry analysis of fractions collected at different flow rates**

| Flow rate (ml/min) | 50 | | 55 | | 60 | | 65 | | 70 | | 75 | |
| --- | --- | --- | --- | --- | --- | --- | --- | --- | --- | --- | --- | --- |
| Fraction number | 1 | 2 | 3 | 4 | 5 | 6 | 7 | 8 | 9 | 10 | 11 | 12 |
| Volume (ml) | 100 | 100 | 100 | 100 | 100 | 100 | 100 | 100 | 100 | 100 | 100 | 100 |
| Cell density  (× 10^5^ cells/ml) | 0.29 | 0.23 | 0.33 | 0.34 | 1.01 | 1.03 | 1.15 | 1.18 | 1.21 | 1.23 | 1.11 | 0.90 |
| Flow cytometry analysis | NO (cell density is too low) | | | | YES | | | | NO (fractions contain a mixed cell population) | | | |

**TABLE S4.** **Cell density of fractions with different collection volumes**

| Fraction number | Volume (ml) | Cell density (× 10^5^ cells/ml) |
| --- | --- | --- |
| 1 | 0 ~ 50 | 1.08 |
| 2 | 50 ~ 100 | 1.14 |
| 3 | 100 ~ 150 | 1.20 |
| 4 | 150 ~ 200 | 1.04 |
| 5 | 200 ~ 250 | 0.82 |
| 6 | 250 ~ 300 | 0.85 |

**References**

Banfalvi, G. (2008). Cell cycle synchronization of animal cells and nuclei by centrifugal elutriation. *Nature protocols* 3(4), 663–673. doi: 10.1038/nprot.2008.34.

Banfalvi, G. (2017). "Synchronization of mammalian cells and nuclei by centrifugal elutriation," in *Cell cycle synchronization. methods in molecular biology (methods and protocols).* ed. G. Banfalvi. (New York: Humana Press), 31–52.

Delgado, M., Kothari, A., Hittelman, W.N., and Chambers, T.C. (2017). Preparation of primary acute lymphoblastic leukemia cells in different cell cycle phases by centrifugal elutriation. *Journal of Visualized Experiments* 2017(129), e56418. doi: 10.3791/56418.
